# Supplementary figures and images for: RNA-binding protein syncrip regulates starvation-induced hyperactivity in adult Drosophila
Source: PLoS Genet. 2021 Feb 22;17(2):e1009396. doi: 10.1371/journal.pgen.1009396 (PMC7932510; doi:10.1371/journal.pgen.1009396)

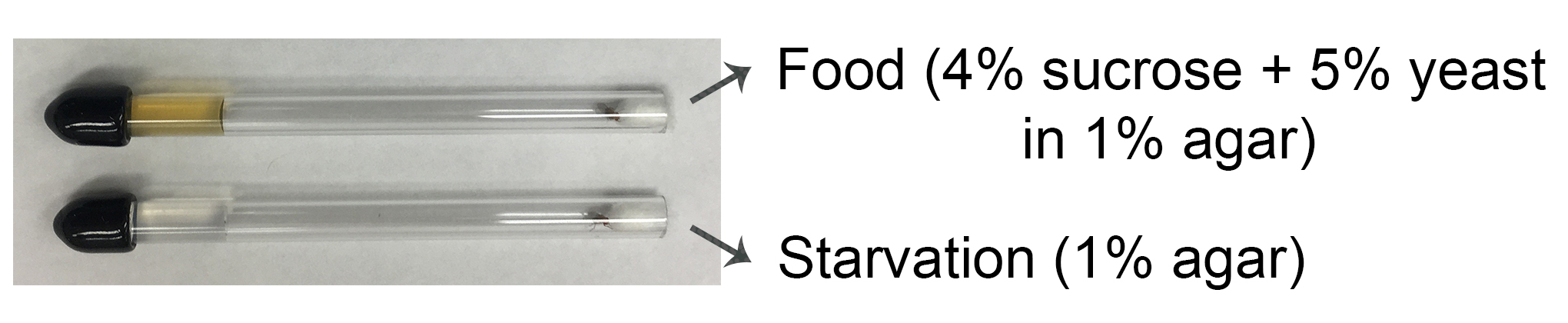

Supplement: S1 Fig — One end of the activity tube (Diameter × Length = 5 mm x 65 mm, Trikinetics Inc.) was filled with either 4% sucrose plus 5% yeast in 1% agar in the food condition or 1% agar only in the starvation condition. The length of the medium is 1 cm. The other end of the tube was stopped with a small cotton ball. One male fly was introduced into each tube. (TIF) [file pgen.1009396.s001.tif]

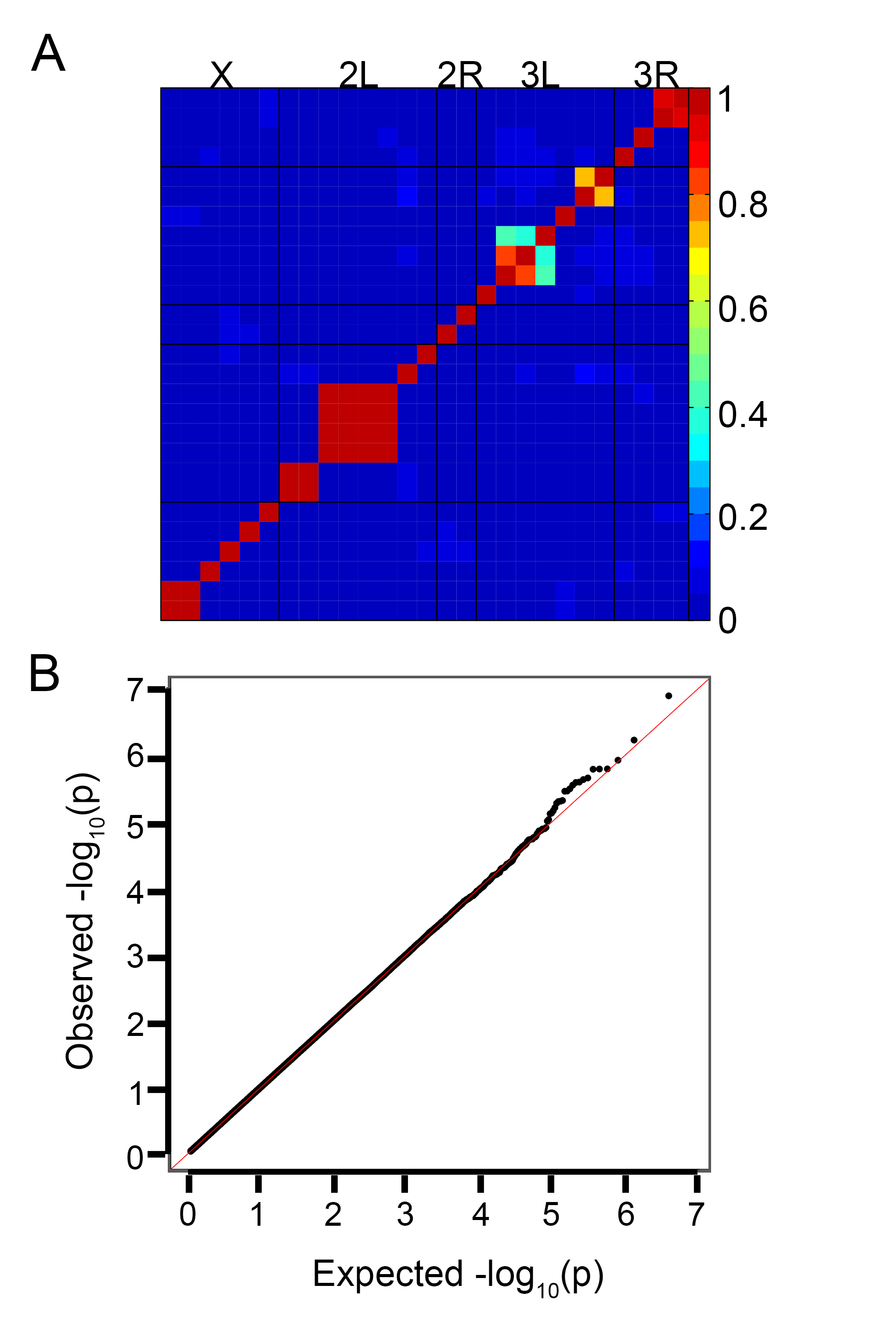

Supplement: S2 Fig — (TIF) [file pgen.1009396.s002.tif]

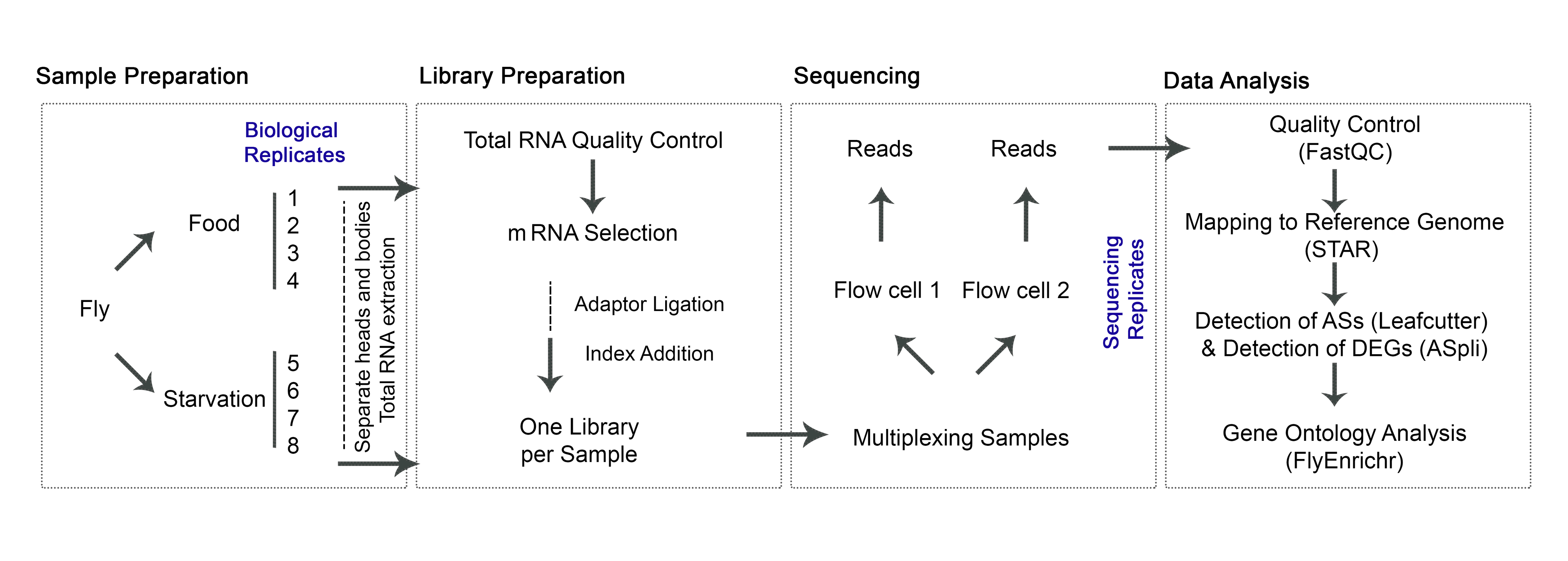

Supplement: S3 Fig — (TIF) [file pgen.1009396.s003.tif]

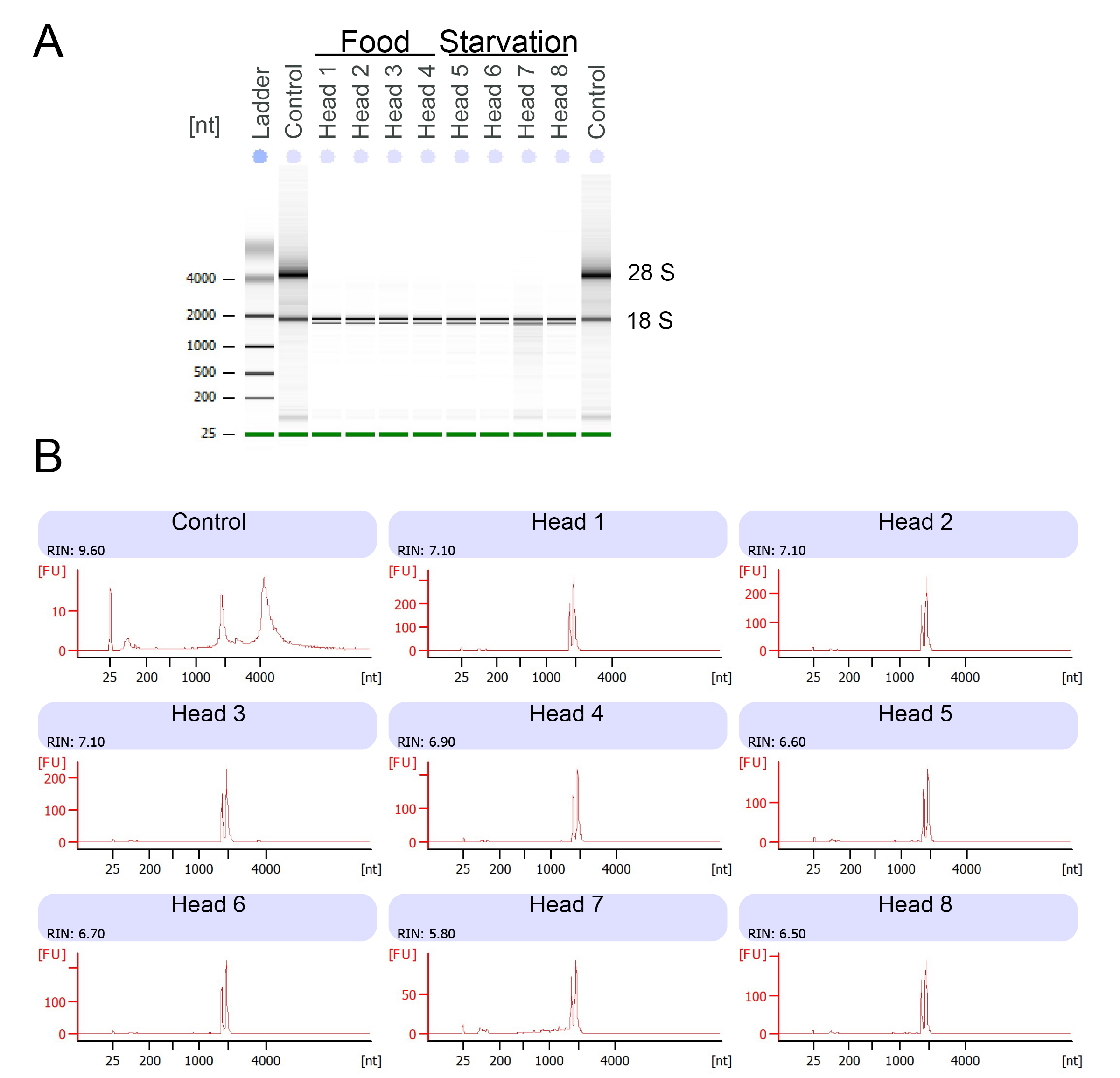

Supplement: S4 Fig — (TIF) [file pgen.1009396.s004.tif]

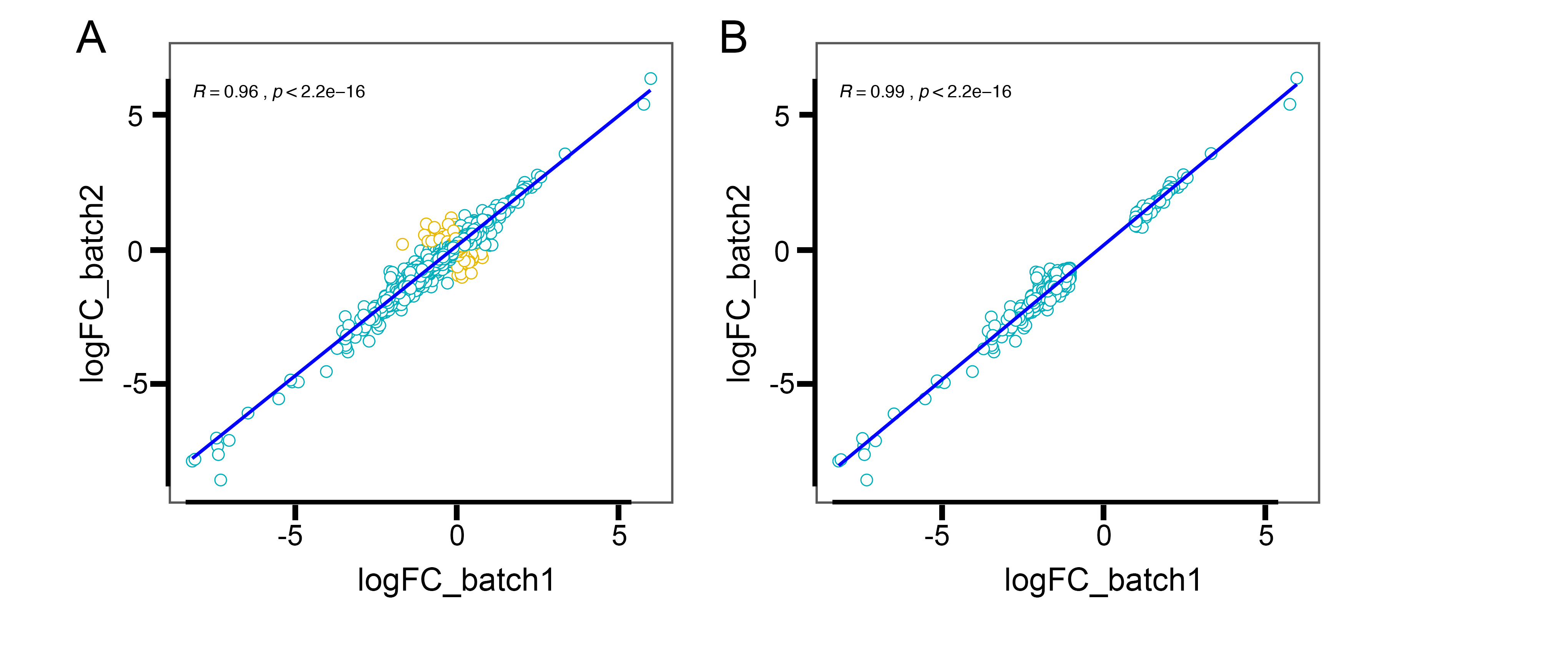

Supplement: S5 Fig — A: Scatter plot for all genes. A total of 9452 genes were included in the analysis. Each dot represents one gene. Dots with cyan color are the genes that show the same direction of change after starvation for two batches. Dots with yellow color are the genes that show the opposite direction of change after starvation for two batches. B: Scatter plot for genes with false discovery rate (FDR) < 0.05 and fold change ≥ 2 or fold change ≤ -2. (TIF) [file pgen.1009396.s005.tif]

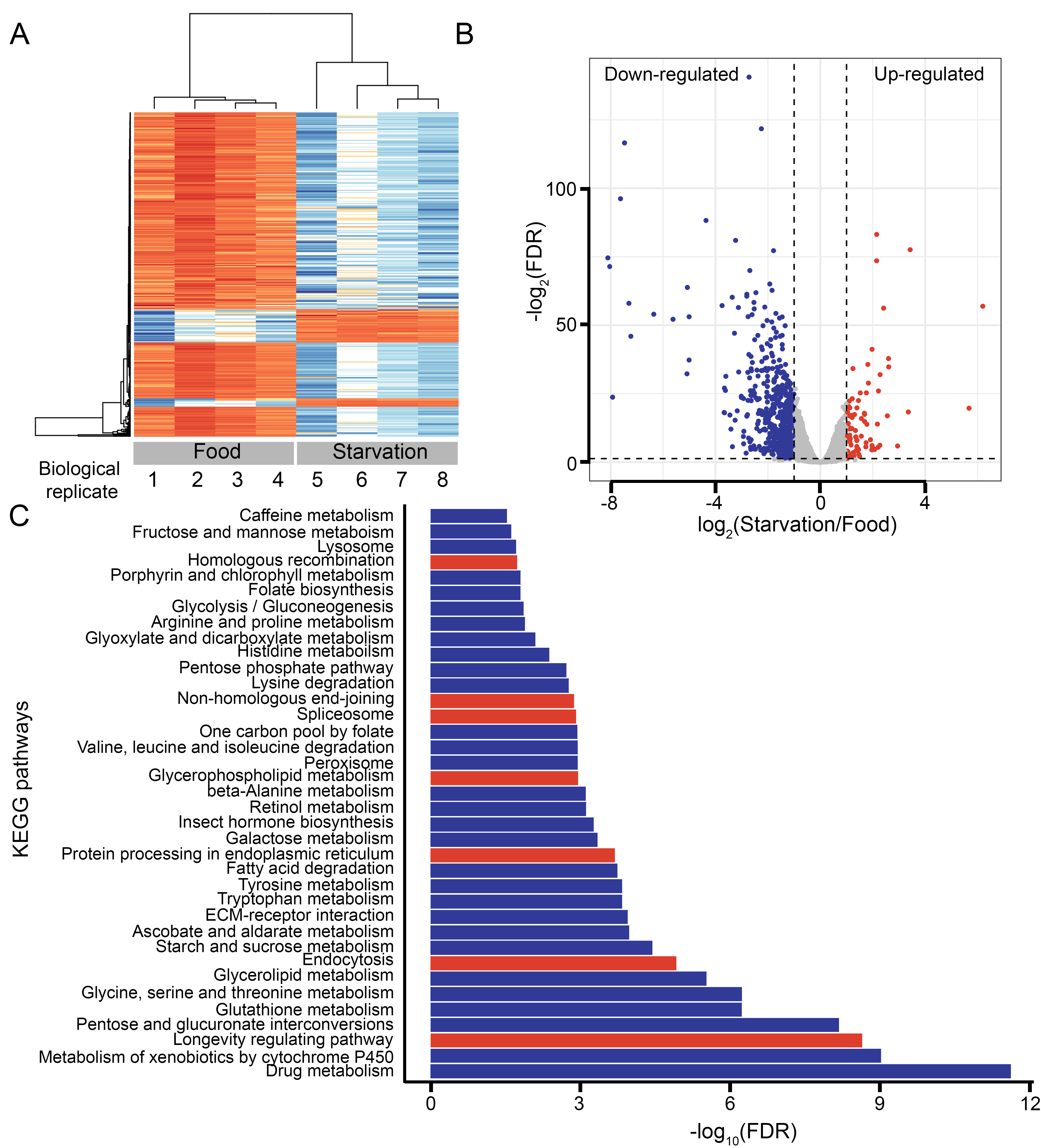

Supplement: S6 Fig — A: Clustering and heatmap showing mRNA levels in four biological replicates of each condition. B: Volcano plot of significantly regulated genes by starvation (Cutoff values: FDR < 0.05 and fold change ≥ 2 or fold change ≤ -2). (C) Enriched KEGG pathways associated with either down-regulated (blue bars) or up-regulated (red bars) genes. (TIF) [file pgen.1009396.s006.tif]

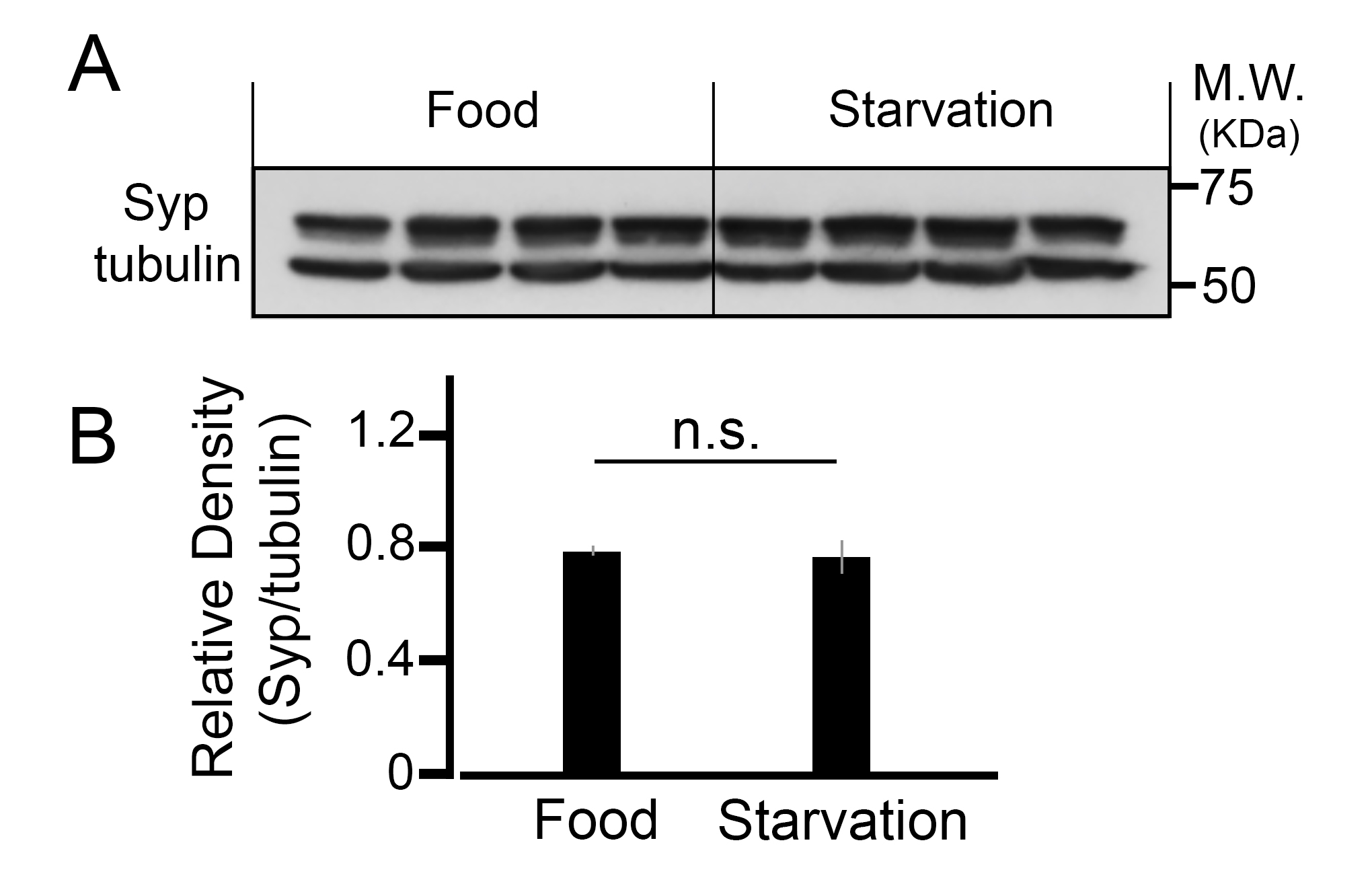

Supplement: S7 Fig — A: Western blot of adult fly head homogenate from w1118 flies under either food or starvation conditions. Four biological replicates per condition. Tubulin is the loading control. B: Quantification of Syp from panel A. n.s: P > 0.05, unpaired t-test. Error bars represent SEM. (TIF) [file pgen.1009396.s007.tif]

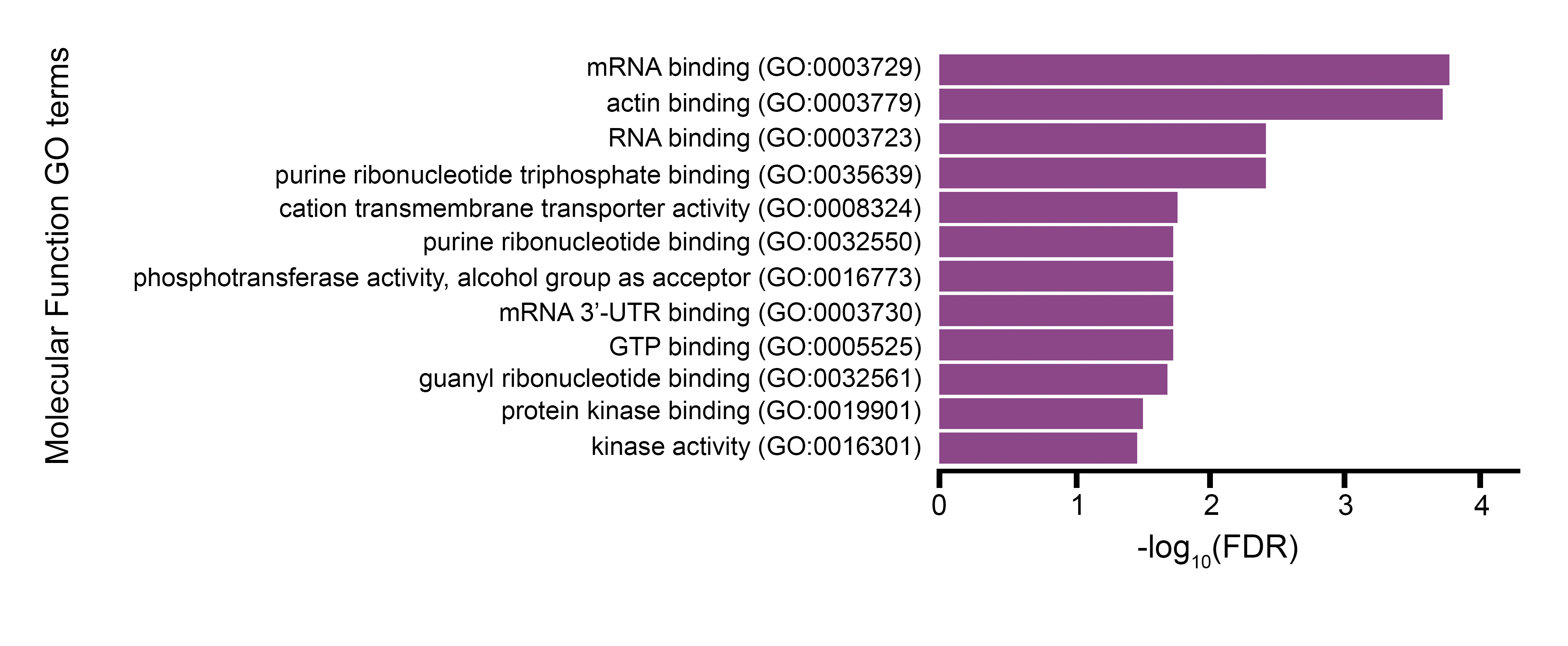

Supplement: S8 Fig — Only terms associated with more than four genes were shown in this plot. See the complete list in S8 Table. (TIF) [file pgen.1009396.s008.tif]
